# A patient-centric approach to chronic rhinosinusitis with nasal polyps (CRSwNP): developing tools to improve disease management and outcomes

**DOI:** 10.1007/s00405-025-09763-5

**Published:** 2025-12-10

**Authors:** Adam M. Chaker, Elena Cantone, Peter W. Hellings, Nathalie Heirman, Benjamin Verillaud, Valerie Hox, Cristina Jacomelli, Joaquim Mullol

**Affiliations:** 1 Department of Otorhinolaryngology and Center for Allergy and Environment (ZAUM), TUM School of Medicine and Health, TUM University Hospital, Klinikum rechts der Isar, Technical University of Munich, Munich, Germany; 2 Department of Neuroscience, Reproductive Sciences and Dentistry, ENT section, University of Naples “Federico II”, Naples, Italy; 3 Laboratory of Allergy and Clinical Immunology Research Unit, Department of Microbiology, Immunology and Transplantation, KU Leuven, Leuven, Belgium; 4 The European Forum for Research and Education in Allergy and Airway Diseases Scientific Expert Team Members, Brussels, Belgium; 5 Laboratory of Upper Airways Research, Department of Otorhinolaryngology, University Hospital Ghent, Ghent, Belgium; 6 Department of otorhinolaryngology, Head and Neck Surgery, UZ Leuven, Leuven, Belgium; 7 GSK, Wavre, Belgium; 8 Service d’ORL et chirurgie cervico-faciale, hôpital Lariboisière, Assistance publique–Hôpitaux de Paris, Paris, France; 9 Université Paris Cité, Paris, France; 10 Inserm U1131, Paris, France; 11 Department of Otorhinolaryngology, Head and Neck Surgery, Cliniques Universitaires Saint-Luc, Brussels, Belgium; 12 Associazione Nazionale Pazienti Respiriamo Insieme – APS, Padova, Italy; 13 Rhinology Unit and Smell Clinic, ENT Department, Hospital Clínic Barcelona, IDIBAPS, Universitat de Barcelona, CIBERES, Barcelona, Catalonia Spain

**Keywords:** Chronic rhinosinusitis with nasal polyps, ENT, Inflammation, Otorhinolaryngology, Patient management plan

## Abstract

**Purpose:**

Chronic rhinosinusitis with nasal polyps (CRSwNP) is an upper airways disease predominantly characterized by type 2 (T2) inflammation, leading to reduced quality of life and patient/healthcare burden. Many patients remain underdiagnosed for T2 inflammation in clinical practice as it is not often defined/considered, resulting in inadequate disease management. This research aimed to develop and evaluate a patient management plan (PMP) to be used by healthcare professionals (HCPs) in clinical practice to improve the management and standardization of management of patients with CRSwNP in Europe.

**Methods:**

A working group of six otorhinolaryngology (ORL) specialists and one patient advocacy group (PAG) representative, who form part of the European CRSwNP Alliance, developed the PMP. Two online surveys were disseminated to ORLs/PAGs and national CRSwNP Alliance members in December 2024 and January 2025, respectively, to evaluate the design and potential utility of the PMP in clinical practice across Europe.

**Results:**

Survey 1 respondents agreed that key themes in the initial PMP (patient symptoms, management of comorbidities, agreed treatment goals, follow-up management) were likely to be effective in improving patient management. Respondents across both surveys suggested that the PMP would improve shared decision-making, follow-up, adherence, and increase understanding of CRSwNP as a chronic inflammatory condition. They agreed that the PMP could standardize the management of CRSwNP across Europe.

**Conclusions:**

The PMP could support patient–HCP education and communication, supplement management guidance, enhance shared decision-making, and ensure a timely, personalized, long-term treatment and management approach, including follow-up, for patients with CRSwNP.

**Supplementary Information:**

The online version contains supplementary material available at 10.1007/s00405-025-09763-5.

## Written plain language summary

Chronic rhinosinusitis with nasal polyps (CRSwNP) is a disease that is also referred to as ‘nasal polyp syndrome’. People who have CRSwNP experience symptoms such as loss of smell, blocked and/or runny nose, difficulty sleeping, tiredness, and pressure/pain across their face. It usually impacts a person’s quality of life. A person with CRSwNP has significant sinus and nasal inflammation which is not always accurately recognized. This means that, occasionally, people do not receive the correct treatment for their disease. The aim of this research was to design and evaluate a patient management plan (PMP) that can be used by physicians with their patients to improve the management of CRSwNP.

The PMP is a document that was designed by ear, nose, and throat specialists, and a representative for patients who were interested in improving the health of people with CRSwNP. The PMP was sent to many specialists across Europe, including lung, allergy, ear, nose, and throat physicians, and patient representatives. They were also sent two surveys to ask what they thought about the PMP. Specialists and patient representatives responded to the surveys based on how they felt the PMP would help physicians and patients in making better treatment decisions. Overall, they felt that the key themes detailed in the PMP (education, early diagnosis, personalized treatment, adherence to treatment, follow-ups, and long-term) were important for managing treatment decisions. They agreed that the PMP would be a useful tool for both physicians and patients. They thought it would help physicians and patients to communicate and make decisions together and improve the long-term management plan for patients. Overall, they thought the PMP could be used with patients with CRSwNP across Europe to improve their treatment process. The final patient-accessible version of the PMP is presented in Supplementary Fig. S1.

## Introduction

### Chronic rhinosinusitis with nasal polyps (CRSwNP)

CRSwNP is a chronic inflammatory disease associated with significant morbidity and disease burden, comparable with that of other chronic diseases including chronic obstructive pulmonary disease, asthma, and diabetes [1]. Available epidemiological data from Spain and Germany suggest that CRSwNP has a prevalence of 0.5–0.6% [2, 3]; approximately 42% of patients with CRSwNP have uncontrolled disease [4].

Over 80% of patients have type 2 (T2) inflammation [1, 5, 6], characterized by elevated levels of T2 cytokines (interleukin [IL]-4, IL-5, and IL-13) that regulate the differentiation and proliferation of eosinophils, leading to their enhanced survival and activation in the sinonasal mucosa [5, 7]. Evidence suggests that biomarkers of T2-driven CRSwNP, particularly eosinophils and IL-5, are strong predictors of recurrent disease following surgery [7, 8]. Identifying and understanding the patient’s individual inflammatory profile can support disease management. Therefore, T2 inflammatory profiling of patients with CRSwNP preoperatively may help to stratify those who are at higher risk of recurrence and may guide clinicians to consider various management [8]. The inflammatory endotype can be identified through various clinical symptoms, measures, and biomarkers [5, 9, 10]. Non-biological markers indicative of T2 inflammation in CRSwNP include increased atopy, olfactory dysfunction, mucus hypersecretion, presence of comorbidities (non-steroidal anti-inflammatory drug-exacerbated respiratory disease [N-ERD] or asthma), and nasal polyp formation [9–11].

Patients with CRSwNP often have a complex disease journey [12], with those in Europe reportedly waiting an average of 21.5 months before seeking support from their healthcare professionals (HCPs) once sinonasal symptoms become unmanageable [11, 12]. There is frequent delayed or misdiagnoses [12]; patients often visit multiple medical specialists prior to treatment and do not undergo nasal endoscopy or smell tests [11–14].

The ultimate treatment goal for patients with CRSwNP is clinical remission (defined as no symptoms, no impact on quality of life [QoL], no requirement for endoscopic sinus surgery [ESS], or chronic/rescue oral corticosteroids [OCS], and recovery of smell function) [15, 16]. Current standard of care includes treatment with intranasal saline lavage and intranasal corticosteroids (INCS). OCS may be used in case of severe exacerbations, ESS (including revision surgery) performed for patients who remain uncontrolled with corticosteroid treatment, and biologics for type 2 inflammation where surgical treatment does not improve symptoms [11, 15, 17, 18]. However, treatment duration, prevention of disease recurrence and short- and long-term adverse effects should be considered, with treatment choice optimized based on the patient’s phenotypic characteristics [11, 19–21].

Recently, there has been growing recognition of CRSwNP as a chronic inflammatory disease. There are currently three approved biologic therapies for CRSwNP in Europe that can suppress T2 inflammation: dupilumab, mepolizumab, and omalizumab [8, 11, 22–24]. However, only a small proportion of patients who may be eligible for biological therapy are considered. The European Forum for Research and Education in Allergy and Airway Diseases (EUFOREA) and the European Position Paper on Rhinosinusitis and Nasal Polyps (EPOS) recommend that biologic treatment is initiated in patients with severe bilateral CRSwNP who have undergone previous ESS, and who meet at least three of the following criteria: biological biomarker evidence of T2 inflammation (tissue eosinophils ≥ 10 cells/hpf or blood eosinophil count ≥ 150 cells/µL or total immunoglobulin E (IgE) ≥ 100 IU/mL; ≥ 2 courses of systemic corticosteroids per year or > 3 months low-dose systemic steroids; significant QoL impairment (Sino-Nasal Outcome Test-22 ≥ 40); significant loss of smell (anosmia by smell test or Visual Analogue Scale); and comorbid asthma requiring regular inhaled corticosteroids [11, 15, 25, 26].

### Unmet need for a patient management plan (PMP) for patients with CRSwNP

International guidelines include the current treatment recommendations and detail the heterogeneity of the many different phenotypes and endotypes of CRSwNP. However, guidelines often do not provide a practical overview of timepoints or guidance on specific actions for management and follow-up beyond initial diagnosis and treatment. Patients may remain undiagnosed or be lost to follow-up in the long-term, making re-entry into the care pathway difficult and leading to an increased burden on the patient, HCP, and healthcare system. Therefore, there is an unmet need for a comprehensive, standardized, easy-to-use PMP that details the long-term treatment management plan options for patients with CRSwNP which target underlying inflammatory mechanisms [20]. The PMP could drive standardization of patient visits and journey, and disease management across Europe. It could also allow for further recognition of the substantial disease and health-related QoL burden that patients with CRSwNP experience and support secondary care practitioners in prescribing novel therapies they may be less familiar with.

**Fig. 1:**
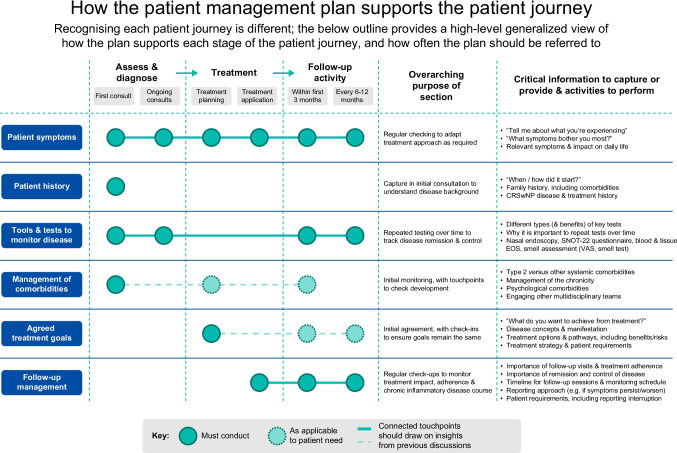
Final version of the PMP. CRSwNP, chronic rhinosinusitis with nasal polyps; EOS, eosinophils; PMP, Patient Management Plan; SNOT-22, Sino-Nasal Outcome Test-22 questionnaire; VAS, Visual Analogue Scale (0–10 cm)

In this paper, an expert group of otorhinolaryngology (ORL) specialists and patient advocacy group (PAG) representatives from Belgium, France, Germany, Italy, and Spain convened with the aim to increase the understanding of the inflammatory, chronic nature of the disease and develop a PMP that could improve long-term disease management and patient experience for CRSwNP in Europe (Fig. 1). This is to be achieved through development, evaluation, and refinement of a PMP to be used as guidance in clinical practice to track individual progress, optimize disease management, and improve patient QoL and outcomes.

## Methods

### Establishing the inflammatory mindset working group

A European CRSwNP Alliance was established in March 2024 and included 17 ORL specialists, three PAG representatives, and two representatives of European Societies (EUFOREA and European Academy of Allergy & Clinical Immunology [EAACI]; Supplementary Fig. S2). From the European CRSwNP Alliance, three regional working groups were created to define, develop, and pilot projects that can be implemented locally to improve the journey for patients with CRSwNP; the Inflammatory Mindset Working Group was one of these three. Members were identified as having direct experience of working with patients with CRSwNP in Europe and had acknowledged that there is a need to challenge current clinical practice to improve patient and treatment outcomes.

### PMP development

The Inflammatory Mindset Working Group met over three virtual meetings in May, June, and September 2024, alongside continued offline communication, to discuss and reach a verbal consensus on key themes of the PMP, including sections with advice for each stage of the patient treatment and management journey, underpinned by scientific evidence. They also discussed which existing validated tools should be referenced within the PMP. They considered the audience, usage, type of advice, content type, level of detail, and thoroughness of the PMP. For each of the themes identified, they discussed: the purpose of each section; what was critical for inclusion; what information would add additional value; and how best to present information for the PMP user. They considered the layout, design, and format as well as legal and compliance considerations including General Data Protection Regulation (GDPR) and digital access. They also considered how to best capture data, ensure consistency between hospital records, and effectively tailor the PMP for individual patients.

### PMP evaluation

For the PMP to be refined, the various components were evaluated through two surveys. Both surveys were developed by the Inflammatory Mindset Working Group to include key items relating to the PMP. Survey 1 sought to assess the utility of the PMP from a European perspective. Survey 2 sought to assess the country-specific utility of the PMP for patients in France, Germany, Italy, and Spain. Where consensus was sought, a five-point Likert-scale (1: strongly disagree to 5: strongly agree; described as mean score out of 5.00) was used; agreement was defined as a Likert scale rating of 4 or 5 and disagreement was defined as a Likert scale rating of 1 or 2. The proportion of respondents in agreement are reported. Where more detailed information was sought, multiple choice or open questions were posed.

### Survey 1: qualitative evaluation of the PMP utility from a European perspective

Survey 1 (Supplementary Table S1), along with a draft version of the PMP, was developed online by a market research agency (Entreprise de Communications Tank Inc.) and disseminated anonymously online via Microsoft Forms to the 17 ORLs and five PAGs, including one EUFOREA, and one EAACI representative, who participated in the European CRSwNP Alliance in December 2024 (Supplementary Fig. S2). Respondents did not receive remuneration. It was estimated to take 10–15 min to complete and aimed to identify areas of the PMP that could be refined. Responses were analyzed descriptively.

### Survey 2: qualitative evaluation of the PMP for country-specific use

Following generally positive feedback from Survey 1, the unabridged PMP was disseminated alongside Survey 2 (Supplementary Table S2) in January 2025 to 42 CRSwNP National Alliance members and representatives from Belgium, France, Germany, Spain and Italy, including four PAGs (one EUFOREA, and one EAACI representative; Supplementary Fig. S2). They were selected based on their expertise in working with patients with CRSwNP and agreed acknowledgement that there is a need to challenge current clinical practice to improve patient and treatment outcomes. Survey 2 was analyzed descriptively, and responses were used to generate the final PMP and provide insights into future utility.

## Results

### Initial design of PMP

Key themes and considerations for inclusion in the PMP are detailed in Table 1. From this, the PMP was consensually developed to include six key sections: (1) patient symptoms; (2) patient history; (3) tools and tests to monitor disease; (4) management of comorbidities; (5) agreed treatment goals; and (6) follow-up management. Each section was articulated to ensure patient-centricity; for example, open-ended questions (‘could you tell me about what you are experiencing?’; ‘what do you want to achieve from your treatment?’) were included to facilitate in-depth, engaging conversations during patient–HCP consultations.

**Table 1:** Key themes considered for inclusion in the Patient Management Plan

Initial themes	Theme aim	Detail per theme (not exhaustive)
Patient history	Defining patient’s phenotype	• Previous ESS • Frequency and dosing of different treatments including use of OCS • Response to previous treatments • Adherence to treatments and lifestyle
Patient symptoms	Understanding the patient disease burden	• Severity/impact of each symptom on the patient • What is being done to address the most impactful symptom, as identified by the patient • Lifestyle/background
Tools and tests to monitor disease	Capture technical information on the patient and build an empathetic understanding of the patient’s situation to better understand their goals and tailor treatment	• Questionnaires to assess symptoms (e.g., VAS, Likert) and impact on QoL (SNOT-22) • Loss of smell by smell test/VAS • Use of clinical and biological biomarkers (e.g., to measure disease severity) • Linking to control/outcomes of standard of care
Assessment of disease control	Capture a baseline, or continue tracking, data to visualize improvement in burden and sinonasal symptoms over time	• Include references to any control tools in existence (as detailed in current treatment guidelines) or in development
Results of previous QoL questionnaires	–	• Consider guidance to implement QoL questionnaires to ensure these are considered in follow-up appointments throughout the patient’s journey and with other specialties
Management of comorbidities	Tailor treatment and disease management according to any other prevalent comorbidities	• Impact on ability to control CRSwNP • Involvement of wider MDT in the patient’s care
Agreed treatment goals	Understand the patient goals to personalize key performance indicators of success and outline expectations to the patient	• Patient and clinician perspectives – including patient eligibility for biologics dependent on country-specific guidance
Follow-up recommendations	Drive consistency in ongoing management and support of patient in disease treatment, including adherence	• Frequency depending on level of disease control; types of tests to perform; patient expectations regarding treatment and how this matched reality; when to consult the ORL specialist or other MDT a. There is no official guidance regarding frequency of follow-ups and most ORL specialists will follow their local reimbursement criteria b. This category can be used as a springboard to discuss chronic, recurring nature of CRSwNP

*CRSwNP* Chronic rhinosinusitis with nasal polyps; *ORL* Otorhinolaryngology; *ESS* Endoscopic sinus surgery; *MDT* Multidisciplinary team; *OCS* Oral corticosteroids; *QoL* Quality of life; *SNOT-22* Sino-Nasal Outcome Test-22 questionnaire; *VAS* Visual Analogue Scale (0–10 cm)

During the development of the PMP, the Inflammatory Mindset Working Group considered its utility in conjunction with existing treatment guidelines. It was agreed that the PMP should be a standalone document and may signpost to other resources, such as EUFOREA and EPOS guidelines, for additional information.

The Inflammatory Mindset Working Group also acknowledged that the PMP would likely need to be available in multiple formats; for example: (1) an app that is completed by the patient/ORL specialist; (2) a digital program that can be accessed on multiple devices (allowing the ORL specialist to complete the information on a tablet and the patient to access on their phone); and (3) a document or website focused on patient education. It was noted that any digital document may have challenges with GDPR, patient access, and integrating into existing systems. The working group also noted that motivating patients and ORL specialists to fill in the PMP, irrespective of the final format, was a concern.

### Survey results

**Fig. 2:**
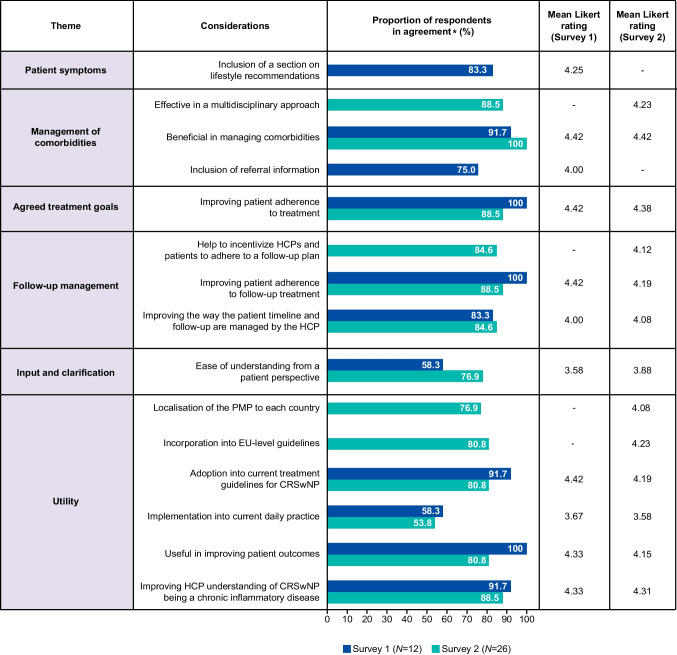
Surveys 1 and 2: PMP feedback from HCP and PAG representatives using Likert scales (0.00–5.00). *In agreement: respondents scoring 4.00 or 5.00 (out of 5.00). CRSwNP, chronic rhinosinusitis with nasal polyps; EU, European Union; HCP, healthcare professional; PAG, patient advocacy group; PMP, Patient Management Plan

**Fig. 3:**
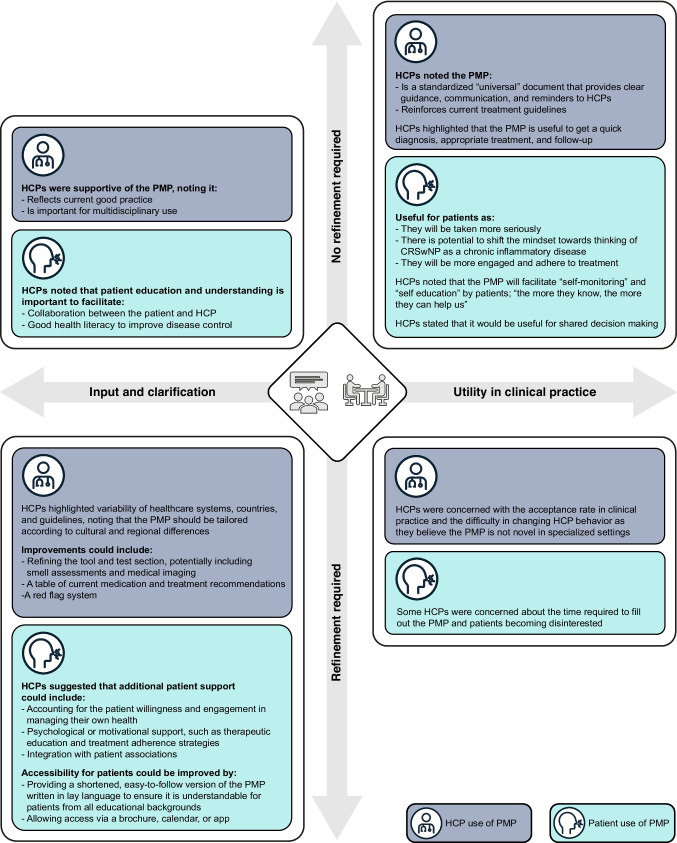
Surveys 1 and 2: PMP feedback from HCP and PAG representatives on input and clarification, and utility. HCP, healthcare professional; PAG, patient advocacy group

Overall, 12 (response rate 54.5%) and 26 (response rate 61.9%) specialists (including ORL specialists and patient representatives) anonymously responded to Survey 1 and Survey 2, respectively; results are presented in Figs. 2 and 3 and Supplementary Tables S1 and S2. Respondents generally agreed that the key themes detailed in the PMP were likely to be effective in improving patient management and increase understanding of the chronic inflammatory nature of CRSwNP.

### Theme 1: patient symptoms

The inclusion of a lifestyle recommendations section in the PMP was agreed by the majority (*n* = 10; 83.3%) of respondents in Survey 1 to be useful (mean Likert 4.25; Fig. 2); however, two respondents (16.7%) were unsure (Likert 3.00; Supplementary Table S1).

### Theme 2: management of comorbidities

Respondents felt that the PMP would be beneficial in managing comorbidities (Survey 1 and 2 mean Likert: 4.42 and 4.42, respectively) and that the inclusion of referral information for comorbidities would be useful (Survey 1 mean Likert only: 4.00; Fig. 2). In Survey 2, most respondents (*n* = 23; 88.5%) felt that the PMP would be effective for multidisciplinary approaches (mean Likert 4.23). When asked to elaborate, many respondents qualitatively stated that it will allow for comprehensive disease evaluation, increase between-specialist communication, and further involve patients (Supplementary Table S2). Of the three who were unsure/disagreed, it was reported that a multidisciplinary approach is difficult to achieve due to differences between specialties and their respective guidelines in different countries.

### Theme 3: agreed treatment goals

Consensus was reached in that the proposed PMP would be effective in improving patient adherence to treatment (Survey 1 and 2 mean Likert: 4.42 and 4.38, respectively; Fig. 2).

### Theme 4: follow-up management

Respondents agreed that the proposed PMP would be effective in improving patient adherence to follow-up treatment (Survey 1 mean Likert: 4.42; Fig. 2) or meetings (Survey 2 mean Likert: 4.19). Overall, respondents agreed that the PMP would effectively improve the way the patient pathways and follow-up consultations are managed by the treating HCP (Survey 1 and 2 mean Likert: 4.00 and 4.08, respectively); however, one Survey 1 respondent (8.3%) and four Survey 2 respondents (15.4%) were unsure/disagreed (Likert 2.00 or 3.00; Supplementary Table S1). Additionally, 22 Survey 2 respondents (84.6%) felt that the PMP would help to incentivize HCPs and patients to adhere to a follow-up plan (mean Likert 4.12), with four being unsure (Likert 3.00; Supplementary Table S2).

### Theme 5: input and clarification

Although many respondents agreed that the proposed PMP would improve overall patient management, they were less sure on how easy it would be for patients to understand the proposed PMP (Survey 1 and 2 mean Likert: 3.58 and 3.88, respectively; Fig. 2). When asked using open-ended questions, Survey 2 respondents suggested that the PMP could be refined and shortened to reduce the time required to complete the sections (Fig. 3). Respondents in both surveys stated that certain information in the proposed PMP was unclear, including accessibility to patients of all educational backgrounds, country variability, guidance for choice of smell assessment, inclusion of medical imaging, and format/implementation of the PMP. Conversely, respondents generally felt that there was no requirement to incorporate or remove any information to/from the PMP, however they agreed that the level of detail in the PMP should also be considered to ensure it is not overwhelming for patients.

### Theme 6: utility

When asked open-ended questions on the utility of the PMP, respondents from both surveys agreed that it would be useful to ensure a standardized, universal approach to improve care and outcomes, alongside optimized adherence and shared decision-making (Fig. 3). They believed that the PMP would facilitate informed consultations and ensure that patients are not lost to follow-up, with one respondent stating that it would be useful “to get a quick diagnosis, appropriate treatment, and consequent follow-up”. Only some respondents believed that the PMP would be easy to implement in current daily practice (Survey 1 and 2 mean Likert: 3.67 and 3.58, respectively; Fig. 2). Particularly, responders qualitatively stated that “the PMP will be variable between setting and healthcare systems” and should be “tailored according to cultural and regional differences, to reflect the healthcare system, country, and guidelines” (Fig. 3). Respondents agreed the PMP would improve patient outcomes and understanding of CRSwNP as a chronic inflammatory disease (Survey 1 and 2 mean Likert: 4.33 and 4.31, respectively) and could be adopted in current treatment guidelines for CRSwNP (Survey 1 and 2 mean Likert: 4.42 and 4.19, respectively; Fig. 2). Respondents agreed that additional support would be required to implement the PMP into clinical practice. The top three key requirements were: (1) an online platform or application; (2) a quick reference guide of the key changes; and (3) detailed written materials outlining the new PMP (Supplementary Fig. S3). All respondents felt that the PMP would improve patient outcomes, compliance, communication, and adherence, and empower patients to make shared decisions about their disease, with one respondent stating the PMP has a “high potential of changing the mindset of both patients and HCPs towards CRSwNP as a chronic inflammatory disease” (Fig. 3). Many believed that the PMP would enhance patient–HCP communication and build trusted relationships in a standardized manner, leading to improved shared decision-making, however some were concerned that it may have low acceptance in clinical practice, particularly at multidisciplinary level.

### Finalization of the PMP

Following evaluation of Survey 1 and 2 results by the European CRSwNP Alliance, it was agreed that no further PMP refinements were required. The final PMP is presented in Fig. 1 and contains detailed information on specific actions and information required for each visit.

## Discussion

Long-term follow-up and management of CRSwNP are highly variable in clinical practice and are not reflective of the heterogeneity and chronicity of the disease [12, 20, 27, 28]. The PMP is hoped to provide a comprehensive guide to ensure a timely, personalized, long-term treatment and management plan to ensure patients are treated according to their individual disease profile.

### The PMP as a key resource in supporting disease management while considering individual patient requirements

It is envisaged that the PMP will be primarily utilized by ORL specialists to consolidate their understanding of CRSwNP, and encourage collaborative discussions and decision-making based on the patients’ immunological disease profiles. With novel knowledge and treatment options (such as biologics) available, HCPs are becoming more aware of the chronic inflammatory nature of CRSwNP and are shifting from phenotype to endotype classification when evaluating patients [29–31]. Nevertheless, translating this knowledge into clinical practice remains an issue, as there is a lack of consensus or standardization on how and when to manage patients based on the endotype of the disease, for example, when to offer ESS, reduce OCS use or initiate biologics [29]. Therefore, there is a need to extend this knowledge further to harmonize understanding, treatment, and management plans [32]. The ‘treatment goals’ theme within the PMP may lead to better treatment decisions, particularly for patients who are eligible for long-term anti-inflammatory treatment and who may also benefit from such treatment not only for CRSwNP but also for other comorbid T2-inflammatory driven diseases, improve adherence to treatment for patients who have severe and chronic disease, understand the adverse events associated with current treatment, and potentially lead to long-term control and remission.

Respondents agreed that the PMP should be easily accessible by patients and well formatted to facilitate completion of relevant sections during consultations while maximizing the educational aspects. In alignment with this, many respondents agreed that it would be beneficial to introduce an online platform, a reference guide, and written materials to facilitate the use of the PMP in clinical practice. However, there are challenges to overcome when implementing the PMP, including patient-, HCP- and service-level barriers. For patients, online platforms may reduce accessibility for those who are not digitally literate or do not have access to the relevant devices [33]. Online accessibility of the PMP may also face GDPR challenges, increasing the need for potential security strategies to ensure data protection, though restricting access of certain elements to specific users may circumvent some of these challenges. Additionally, the average reading level and health literacy of patients, which, along with the possibility of fatigue when completing multiple sections of the PMP, may impact the usability and accuracy of patient responses. These barriers must therefore be considered and adapted to ensure the utility of the PMP is optimized in clinical practice.

### Alignment of the PMP with existing standard of care and current guidelines, and implementation into clinical practice

The PMP was designed to provide further clarity on the management of patients with CRSwNP, from initial consultation and diagnosis through to treatment and long-term follow-up activity, noting current treatment guidance [11, 15]. Many attempts to streamline the guidelines for patient management of CRSwNP have previously been made. For example, in Spain, the POLIposis Nasal 2.0 (POLINA 2.0) was developed to translate current knowledge of pathophysiology, disease burden, diagnosis treatment, and follow-up, into an evidence-based, easy-to-read practical guide for HCPs [34]. Many unmet needs were identified, including the need to define clinical predictors of disease control and make informed, streamlined decisions on the choice of therapy. To address these unmet needs, shared decision-making, alongside patient communication and education, were identified as key solutions that are yet to be optimized in clinical practice [34]. Recently, the Nasal Polyp Patient Assessment Scoring Sheet was developed as an easy-to-use guidance tool, integrating both subjective and objective measures to evaluate patients with CRSwNP [35]; additionally, the EUFOREA patient portal aimed to simplify complex medical concepts for patients with CRSwNP by offering evidence-based resources as well as tips and tricks [36]. The PMP is intended to complement, not replace, existing guidelines by reinforcing and optimizing current management strategies. It will serve as a standardized, structured document that provides guidance, communication, and reminders to HCPs across Europe to support every stage of the patient journey. The concise PMP document outlines proposed actions for each visit and follow-up, helping HCPs create plans for their patients. It is envisaged that the dissemination of the PMP will be the responsibility of the CRSwNP National Alliances; they will translate and validate the PMP and will interact with a variety of disciplines to encourage utilization in clinical practice. As such, it is hoped that the PMP evolves into a key resource that is utilized by many HCPs across Europe to standardize management in clinical practice and facilitate dissemination of the European treatment guidelines.

### Patient education, empowerment, and shared decision-making

Although developed to aid HCP treatment decisions in clinical practice, patients should also be encouraged to use the PMP to better educate and empower themselves to support patient–HCP communication and shared decision-making. The inclusion of a plain language summary (Supplementary Fig. S1) aims to increase patient understanding, improve patient health literacy, and greater facilitate patient–HCP communication and decision-making. Currently, the treatment decision-making process is influenced by a combination of factors, including HCP opinions, personal treatment goals and expectations, treatment availability, regional/national management policies, healthcare systems, and comorbidities [37]. Introducing a multidisciplinary team approach and shared decision-making process may optimize patient–HCP education and communication, refine treatment guidance, and ensure a timely, personalized, long-term treatment and management plan for patients with CRSwNP. Though this approach is ideal in theory, Survey 2 respondents generally felt that incorporation into a multidisciplinary setting may prove difficult. However, in a recent review article, it was reported that 50% of patients with CRSwNP do not have ownership of their treatment plan, 25–30% have some ownership, and 10% are knowledgeable and involved [37]. It is therefore essential to improve a patient’s health literacy, advocate for increased patient ownership in the treatment and management decision-making process, and reduce decisional conflict between long-term treatment pathways [14]. Interestingly, it has been shown that the incorporation of decision aids into clinical practice for various diseases have served to improve shared decision-making between patients and HCPs [38]. As such, the PMP may be a useful aid in CRSwNP to advocate for shared decision-making, patient health literacy, and stimulate active patient participation, which may lead to improved patient outcomes and the efficient management of CRSwNP in clinical settings. Finally, it could increase patient and HCP understanding of CRSwNP as a chronic inflammatory disease and support the identification of inflammatory endotypes, leading to optimal treatment and management decisions.

### Strengths and limitations of this study

A key strength of this study is the formation of an Alliance of Europe-based ORL specialists and PAG representatives with expertise in CRSwNP to develop a PMP that can enhance patient-centric care and improve patient management and outcomes. The Inflammatory Mindset Working Group sought to enhance care through combining new guidance with existing validated tools. The PMP was refined based on feedback from a broader group of specialists and PAG representatives, ensuring that the patient perspective was considered. The innovative nature of the PMP provides guidance to both patients and HCPs on what to assess during consultations. In addition to the current treatment guidelines, the PMP provides clear recommendations and education on treatment decisions based on the inflammatory nature of CRSwNP.

A limitation of this study is that the included ORL specialists and survey respondents were only from select countries, therefore other country-specific considerations may not have been highlighted. Additionally, the PMP was only developed by six ORL specialists and one PAG representative, and its utility was not tested in a patient population; however, the included specialists represented several European countries and all had direct experience in managing patient care, including one PAG who advocated for patients and ensured patient-centricity. Furthermore, consensus was only reached verbally utilizing a pre-hoc process and did not employ a standardized methodology, such as a Delphi consensus study; despite this, the majority of the survey respondents agreed that the key themes of the PMP were likely to be effective in improving patient management, which is in alignment with opinions of the Inflammatory Mindset Working Group. As such, this current proposal of a consented PMP should be evaluated for utility among HCPs and patients across Europe.

## Conclusions

A standardized PMP, with a focus on patient-centric care and a deeper understanding of the inflammatory nature of CRSwNP, could help to enhance the patient journey by improving timely diagnosis and treatment initiation, standardizing follow-up management, optimizing patient ownership and health literacy, and reducing decisional conflict through the incorporation of patient–HCP discussions. By consolidating and providing information to further support patient and HCP understanding and decisions, the PMP could optimize disease management through enhanced identification and treatment of disease severity, chronicity, and control, with the aim to support clinical remission in patients with CRSwNP, supported by national and international consensus and guidelines.

## Supplementary Information

Below is the link to the electronic supplementary material.

Supplementary file1 (DOCX 1091 KB)

## Data Availability

Data are available upon request from the corresponding author.
