# Supplementary material for: A patient-centric approach to chronic rhinosinusitis with nasal polyps (CRSwNP): developing tools to improve disease management and outcomes
Source: Eur Arch Otorhinolaryngol. 2025 Dec 10;283(1):249–61. doi: 10.1007/s00405-025-09763-5 (PMC12904956; doi:10.1007/s00405-025-09763-5)
Supplement: Supplementary file 1 — Supplementary file1 (DOCX 1091 KB) [file 405_2025_9763_MOESM1_ESM.docx]

# Supplementary materials

**Title:** A patient-centric approach to chronic rhinosinusitis with nasal polyps (CRSwNP): developing tools to improve disease management and outcomes

**Journal:** European Archives of Oto-Rhino-Laryngology

**Authors:** Adam M. Chaker, Elena Cantone, Peter W. Hellings, Nathalie Heirman, Benjamin Verillaud, Valerie Hox, Cristina Jacomelli, Joaquim Mullol

**Corresponding author:** Adam Chaker, [adam.chaker@tum.de](mailto:adam.chaker@tum.de), Department of Otorhinolaryngology and Center for Allergy and Environment (ZAUM), TUM School of Medicine and Health, TUM University Hospital, Klinikum rechts der Isar, Technical University of Munich, Germany

# Supplementary Table S1 Survey 1 questions and results

|  | **Number** | **Question** | **Response type** | **Responses (*N*=12)** | | | | | |
| --- | --- | --- | --- | --- | --- | --- | --- | --- | --- |
|  |  |  |  | **Likert score, *n* (%)** | | | | | **Mean Likert** |
|  |  |  |  | 1 | 2 | 3 | 4 | 5 |  |
| Patient symptoms | 1 | How useful do you think it is having a section on lifestyle recommendations included in the management plan? | Five-point Likert scale  (not at all useful 🡪 highly useful) | 0 | 0 | 2  (16.7) | 5 (41.7) | 5  (41.7) | 4.25 |
| Comorbidities | 2. | How beneficial do you think the PMP would be for managing comorbidities? | Five-point Likert scale  (not at all beneficial 🡪 highly beneficial) | 0 | 0 | 1  (8.3) | 5 (41.7) | 6  (50.0) | 4.42 |
|  | 3. | How useful do you find the inclusion of referral information in the management plan? | Five-point Likert scale  (not at all useful 🡪 highly useful) | 0 | 1  (8.3) | 2  (16.7) | 5 (41.7) | 4  (33.3) | 4.00 |
| Agreed treatment goals | 4. | How effective do you think the proposed management plan would be in improving patient adherence to treatment? | Five-point Likert scale  (not at all effective 🡪 highly effective) | 0 | 0 | 0 | 7 (58.3) | 5  (41.7) | 4.42 |
| Follow-up management | 5. | How effective do you think the proposed patient management plan would be in improving patient adherence to follow-up treatments? | Five-point Likert scale  (not at all effective 🡪 highly effective) | 0 | 0 | 0 | 7 (58.3) | 5  (41.7) | 4.42 |
|  | 6. | To which degree would the PMP improve the way the patient timeline and follow-up consultation are managed by the treating HCP? Comparing it to current  management/systems, where follow-up session are not routine. | Five-point Likert scale  (not at all improve 🡪 highly improve) | 0 | 1  (8.3) | 1  (8.3) | 7 (58.3) | 3  (25.0) | 4.00 |
| Input and clarification | 7. | From a patient perspective, how easy would it be to understand and follow the proposed patient management plan? | Five-point Likert scale  (very difficult 🡪 very easy) | 0 | 1  (8.3) | 4  (33.3) | 6 (50.0) | 1  (8.3) | 3.58 |
|  | 8. | Is there anything that seems unclear? | Open field | *“I think the "thing" is not clear to everyone. What will it be exactly, a piece of paper, a booklet, an electronic document or app?”*  *“I think, the plan is too confusing and not understandable for patients from all educational backgrounds”*  *“The PMP will be variable between setting and healthcare systems”* | | | | | |
|  | 9. | Is there anything that should be added? | Open field | *“A kind of table of current medication at every visit (if there will be a page for every visit) and a treatment recommendation or agreed action point (based on mutually agreed expectations)”*  *“Suggestion: visualization of treatment goals / expectations for [treatment example]*; "what can you expect", circle chart with 4 or more symptoms”*  *“The action plan must account for patients' willingness to manage their health, as those needing closer monitoring are often the least engaged. While multidisciplinary (e.g., allergology, pneumology) is well addressed, mental health is overlooked. Psychological or motivational support, such as therapeutic education, treatment adherence strategies, or patient associations, should be integrated”* | | | | | |
|  | 10. | Is there anything that should be taken out? | Open field | *“In some countries, waiting list for [ORLspecialist]* clinics are up to 18 months. In that case, it can be unrealistic to have several appointments a year”*  *“For engaged and discerning patients, it is a good plan, but it might be overwhelming for patients with lower levels of education”*  *“No”* | | | | | |
| Utility | 11. | Why would the PMP be useful in your daily work with patients? | Open field | *“It is a kind of standardized document that provides guidance but can be used like a worksheet or check-list for both.”*  *“Universal guidance. there are so many different ways of doing things, so many different consultation times, so many different ways of organising and caring for patients, that we need to standardise all that.”*  *“Optimal adherence, shared decision-making and prolonging”*  *“To get a quick diagnosis, appropriate treatment and consequent follow-up”* | | | | | |
|  | 12. | How easy is it to implement the proposed patient management plan in your current daily practice? | Five-point Likert scale  (very difficult 🡪 very easy) | 0 | 0 | 5  (41.7) | 6 (50.0) | 1 (8.3) | 3.67 |
|  | 13. | To implement the proposed management plan, what kind of support would you need? | Multiple choice | 1. An online platform or app to facilitate implementation of the patient management plan (e.g., patient tracking, reminders, educational resources); *n*=8 2. A quick reference guide of the key changes, *n*=7 3. Detailed written materials (e.g., guidelines, protocols) outlining the new patient management plan, *n*=5 4. Recorded training videos or webinars that I can access at my convenience, *n*=5 5. Integration with my existing patient records systems, *n*=5 6. Live online training sessions for me and my staff, with opportunities for Q&A, *n*=3 7. Templates or pre-populated forms for patient communication and documentation, *n*=3 8. In-person training sessions or workshops, *n*=2 9. I would not need any support to implement the proposed patient management plan, *n*=0 | | | | | |
|  | 14. | Do you believe the proposed patient management plan will improve patient outcomes? | Five-point Likert scale  (no, not at all 🡪 yes, to a high degree) | 0 | 0 | 0 | 8 (66.7) | 4 (33.3) | 4.33 |
|  | 15. | Can you elaborate why/why not? | Open field  (based on previous response) | *“It will give patients full control of information and [physicians]* a grid to provide better treatment. It should be considered, that in the beginning it will be time consuming”*  *“As soon as patients feel that they are being taken care of as a whole, they concentrate better on their care and are more convinced that what they are doing with the [physician]* who has done all this for them is good”*  *“Standardization in management involving the patients' needs (and responsibility as well)”* | | | | | |
|  | 16. | How effective do you think the proposed patient management plan would be in improving HCP understanding of CRSwNP being a chronic inflammatory disease? | Five-point Likert scale  (not at all effective 🡪 highly effective) | 0 | 0 | 1 (8.3) | 6 (50.0) | 5 (41.7) | 4.33 |
|  | 17. | Overall, could the proposed patient management plan be adopted in treatment guidelines for CRSwNP? | Five-point Likert scale  (no, not at all 🡪 yes, to a high degree) | 0 | 0 | 1 (8.3) | 5 (41.7) | 6 (50.0) | 4.42 |

*All quotes are verbatim, those denoted with square brackets have been amended to align with terminology used throughout the paper, i.e. ‘ENT’ has been amended to ‘ORL specialist’; ‘doctor’ has been amended to ‘physician’.

CRSwNP, chronic rhinosinusitis with nasal polyps; ENT, ear, nose, and throat; ORL otorhinolaryngology; HCP, healthcare professional; PMP, Patient Management Plan; Q&A, questions and answers.

# Supplementary Table S2 Survey 2 questions and results

| **Theme** | **Number** | **Question** | **Response type** | **Responses (*N*=26)** | | | | | |
| --- | --- | --- | --- | --- | --- | --- | --- | --- | --- |
|  |  |  |  | **Likert score, n (%)** | | | | | **Mean Likert** |
|  |  |  |  | 1 | 2 | 3 | 4 | 5 |  |
| Respondent characteristics | 1. | Which country do you practice in? | Multiple choice | 1. Spain, *n*=7 2. Germany, *n*=7 3. Italy, *n*=6 4. France, *n*=3 5. Belgium, *n*=2 6. Other, *n*=1 | | | | | |
|  | 2. | Where do you practice? | Multiple choice | 1. University hospital, *n*=18 2. Other, *n*=6 3. Local hospital, *n*=2 | | | | | |
|  | 3. | For how many years have you practiced? | Open field | 1. 0–10, *n*=3 2. 11–20, *n*=10 3. 21–35, *n*=13 | | | | | |
|  | 4. | Is there a dedicated rhinology clinic, where you practice? | Multiple choice | 1. Yes, *n*=21 2. No, *n*=5 | | | | | |
|  | 5. | Is there a multidisciplinary network present where you practice? | Multiple choice | 1. Yes, *n*=22 2. No, *n*=4 | | | | | |
| Comorbidities | 6. | How beneficial do you think the PMP would be for managing comorbidities? | Five-point Likert scale  (not at all beneficial 🡪 highly beneficial) | 0 | 0 | 0 | 15 (57.7) | 11 (42.3) | 4.42 |
| Agreed treatment goals | 7. | How effective do you think the proposed management plan would be in improving patient adherence to treatment? | Five-point Likert scale  (not at all effective 🡪 highly effective) | 0 | 0 | 3 (11.5) | 10 (38.5) | 13 (50.0) | 4.38 |
| Follow-up management | 8. | How effective do you think the proposed patient management plan would be in improving patient adherence to follow-up meetings? | Five-point Likert scale  (not at all effective 🡪 highly effective) | 0 | 1 (3.8) | 2 (7.7) | 14 (53.8) | 9 (34.6) | 4.19 |
|  | 9. | To which degree would the PMP improve the way the patient timeline and follow-up consultation are managed by the treating HCP? Comparing it to current management/systems, where follow-up session are not routine. | Five-point Likert scale  (not at all improve 🡪 highly improve) | 0 | 1 (3.8) | 3 (11.5) | 15 (57.7) | 7 (26.9) | 4.08 |
| Input and clarification | 10. | From a patient perspective, how easy would it be to understand and follow the proposed patient management plan? | Five-point Likert scale  (very difficult 🡪 very easy) | 0 | 2 (7.7) | 4 (15.4) | 15 (57.7) | 5 (19.2) | 3.88 |
|  | 11. | Please elaborate your previous answer: Which aspects would make it easier or not easier to understand the PMP? | Open field | *“The PMP is clear and complete, but following or being adherent to this plan is not easy for many patients due to the needed time investment and the improvement already achieved”*  *“The patient needs to understand his/her condition to gain control”*  *“I see the biggest problem in the low acceptance in clinical practice”*  *“It´s clear and short items, easy to understand the implications on the follow up patient”*  *“More the patient knows...more the patient can collaborate”*  *“The PMP must be made available to the patient upfront, ideally as a brochure, calendar or app”*  *“It needs to be in lay language and NOT TOO LONG”* | | | | | |
| Follow-up management | 12. | To what extent will the PMP help incentivize HCPs and patient to adhere to a follow-up plan? | Five-point Likert scale  (not at all 🡪 high degree) | 0 | 0 | 4 (15.4) | 15 (57.7) | 7 (26.9) | 4.12 |
| Comorbidities | 13. | How effective do you think the PMP would be in the multidisciplinary approach? | Five-point Likert scale  (not at all 🡪 highly) | 0 | 1 (3.8) | 2 (7.7) | 13 (50.0) | 10 (38.5) | 4.23 |
|  | 14. | Please elaborate your previous answer | Open field | *“There already are a number of Management Plans raised by a number of Scientific Societies and HCP Consensus from different specialities and not all of them are going in the same direction. HCPs usually use the Management Plans raised by their speciality consensus/guidelines. A multidisciplinary approach, and common to different countries, is always quite difficult to achieve”*  *“Multidisciplinary is not easily achieved. Even though necessity dictates active implementation not all clinics and clinicians will promptly complying even though we have a guideline”*  *“Structured PMP would include multidisciplinary examinations and will strengthen multidisciplinary and a more comprehensive way to look for the disease”*  *“We expect it to have a major impact that will benefit patients and save time for medical professionals”*  *“If the patient knows what to look for, then it will be easier to detect comorbidities”* | | | | | |
| Input and clarification | 15. | Is there anything that seems unclear? | Open field | *“No”*  *“The PMP seems clear to me”*  *“The part on multidisciplinary is very superficial”*  *“The PMP reflects current good practices, for some centres this is not novel at all. For others less specialised groups the PMP could help!”*  *“I am worried about the tools and tests section which is too detailed”* | | | | | |
| Utility | 16. | Why would the PMP be useful in your daily work with patients? | Open field | *“This PMP will be helpful only if reinforcing previous consensus / guidelines and not considered an independent Management Plan”*  *“Because it increases adherence, treatment optimization and prevents flares of the disease”*  *“Clear communication of a PMP will help to arrange appointments and plan visits, for the patients and for the staff”*  *“The PMP is in line what we already do, so it won’t improve nor change anything”*  *“Will facilitate "self-monitoring" for patients and "self-education"*  *“To homogenize the patient treatment. To give patients some stability, when they are diagnosed with a chronic illness, they should feel that there is a clear path for them”* | | | | | |
|  | 17. | How easy is it to implement the proposed patient management plan in your current daily practice? | Five-point Likert scale  (very difficult 🡪 very easy) | 1 (3.8) | 0 | 11 (42.3) | 11 (42.3) | 3 (11.5) | 3.58 |
|  | 18. | To implement the proposed management plan, what kind of support would you need? | Multiple choice | 1. An online platform or app to facilitate implementation of the patient management plan (e.g., patient tracking, reminders, educational resources), *n*=18 2. A quick reference guide of the key changes, *n*=18 3. Detailed written materials (e.g., guidelines, protocols) outlining the new patient management plan, *n*=16 4. Recorded training videos or webinars that I can access at my convenience, *n*=13 5. Templates or pre-populated forms for patient communication and documentation, *n*=12 6. Integration with my existing patient records systems, *n*=12 7. In-person training sessions or workshops, *n*=9 8. Live online training sessions for me and my staff, with opportunities for Q&A, *n*=7 9. I would not need any support to implement the proposed patient management plan, *n*=0 | | | | | |
|  | 19. | Here is the same list of options for support. Please choose the three that would help you the most? | Multiple choice | 1. Detailed written materials (e.g., guidelines, protocols) outlining the new patient management plan, *n*=15 2. A quick reference guide of the key changes, *n*=13 3. An online platform or app to facilitate implementation of the patient management plan (e.g., patient tracking, reminders, educational resources), *n*=13 4. Integration with my existing patient records systems, *n*=10 5. Templates or pre-populated forms for patient communication and documentation, *n*=8 6. Recorded training videos or webinars that I can access at my convenience, *n*=5 7. In-person training sessions or workshops, *n*=4 8. Live online training sessions for me and my staff, with opportunities for Q&A, *n*=2 9. I would not need any support to implement the proposed patient management plan, *n*=0 | | | | | |
|  | 20. | Do you believe the proposed patient management plan will improve patient outcomes? | Five-point Likert scale  (no, not at all 🡪 yes, to a high degree) | 0 | 0 | 5  (19.2) | 12 (46.2) | 9 (34.6) | 4.15 |
|  | 21. | Why yes or why not? | Open field  (based on previous response) | *“Theoretically the proposed PMP may be considered a good tool. However, to implement and disseminate this PMP in daily clinical practice won't be easy, mainly at multidisciplinary level”*  *“The PMP will improve the journey of the patient and make easy the goals of the management. In addition, improve the patient adherence to the treatment”*  *“We already have such a plan in place, and it simplified many procedures for the staff and the patients”*  *“It has a high potential of changing the mindset of both patients and HCPs towards CRS and accepting it as one of the chronic inflammatory diseases”*  *“I hope it will improve patient outcomes but I see two limits: disinterest of some patients and perceived time wasting for [ORL specialists]*”*  *“Yes, if it is used, but we know how difficult it is to motivate a change of behaviour among HCPs”* | | | | | |
|  | 22. | How effective do you think the proposed patient management plan would be in improving HCP understanding of CRSwNP being a chronic inflammatory disease? | Five-point Likert scale  (not at all effective 🡪 highly effective) | 0 | 1 (3.8) | 2 (7.7) | 11 (42.3) | 12 (46.2) | 4.31 |
|  | 23. | Overall, could the proposed patient management plan be adopted in treatment guidelines for CRSwNP? | Five-point Likert scale  (no, not at all 🡪 yes, to a high degree) | 0 | 0 | 5 (19.2) | 11 (42.3) | 10 (38.5) | 4.19 |
|  | 24. | Does the PMP need to be incorporated in EU-level guidelines, for it to be effective? | Five-point Likert scale  (no, not at all 🡪 yes, to a high degree) | 0 | 1 (3.8) | 4 (15.4) | 9 (34.6) | 12 (46.2) | 4.23 |
|  | 25. | To what extent do you believe the PMP contents will need to be localized to each country? | Five-point Likert scale  (no, not at all 🡪 yes, to a high degree) | 0 | 2 (7.7) | 4 (15.4) | 10 (38.5) | 10 (38.5) | 4.08 |
|  | 26. | What is the one thing we can add to the PMP to make it truly effective? | Open field | *“The PMP should be quick and easy to use while definitively being a support of previous guidelines used at national (country specific) and international level (EUFOREA-EPOS)”*  *“Already very good”*  *“Currently overloaded with information”*  *“There are some cultural differences among the countries that can be taken into account”*  *“Make it available to patients from begin - they will know to choose a [physician]* that enables them to achieve optimal results”*  *“Maybe a summary for patients allowing to show them improvement in their condition, or reminding their expectations (I want to sleep well...) related to their  follow-up”* | | | | | |

* All quotes are verbatim, those denoted with square brackets have been amended to align with terminology used throughout the paper, i.e. ENT’ has been amended to ‘ORL specialist’; ‘doctor’ has been amended to ‘physician’.

CRS, chronic rhinosinusitis; CRSwNP, chronic rhinosinusitis with nasal polyps; ENT, ear, nose, and throat; ORL, otorhinolaryngology; EPOS, European Position Paper on Rhinosinusitis and Nasal Polyps; EU, European Union; EUFOREA; European Forum for Research and Education in Allergy and Airway Diseases; HCP, healthcare professional; PMP, Patient Management Plan; Q&A, question and answer.

# Supplementary Fig. S1. Improving the care for people living with CRSwNP


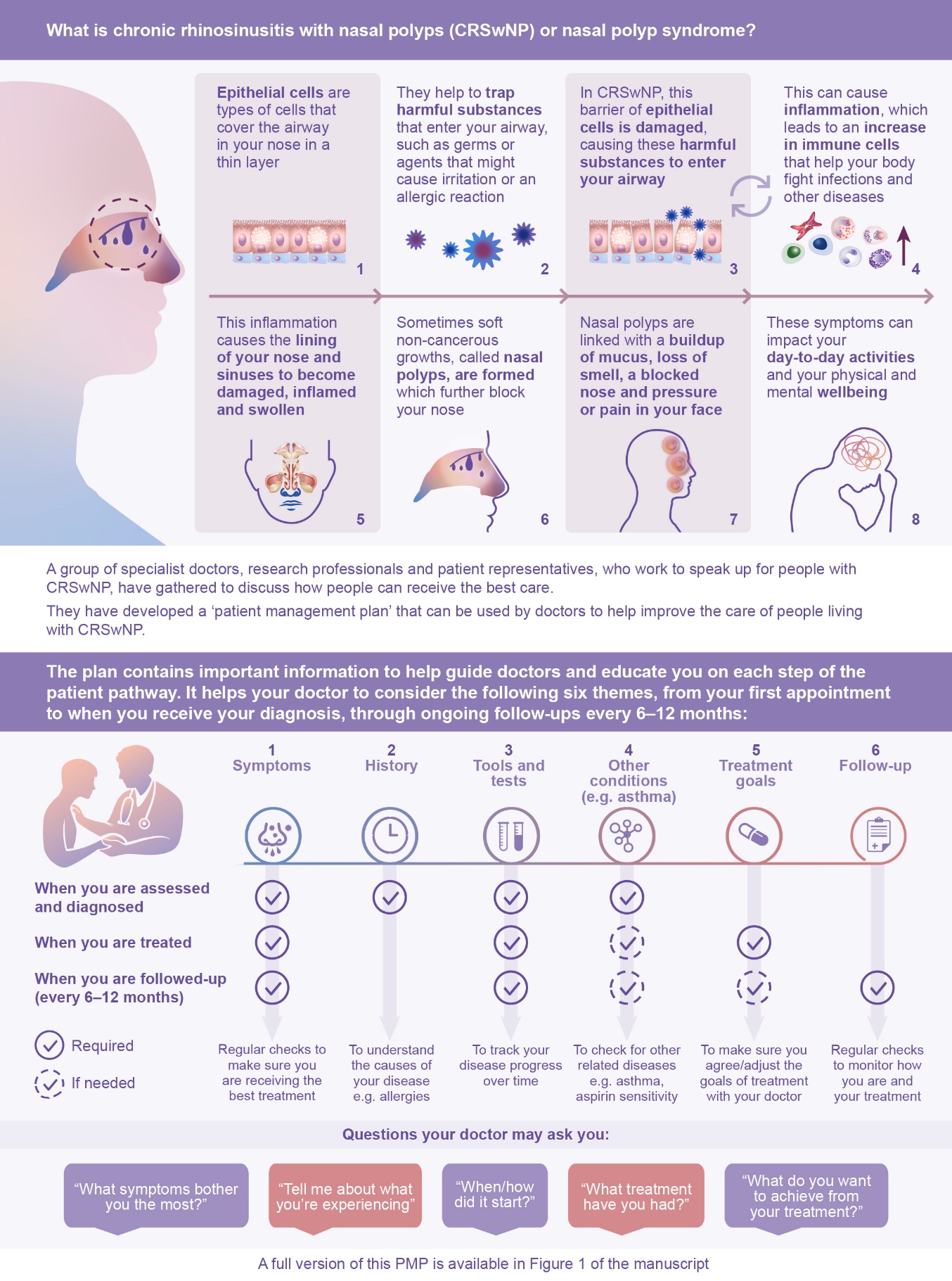


CRSwNP, chronic rhinosinusitis with nasal polyps.

# Supplementary Fig. S2 Formation of the European CRSwNP Alliance and working groups.


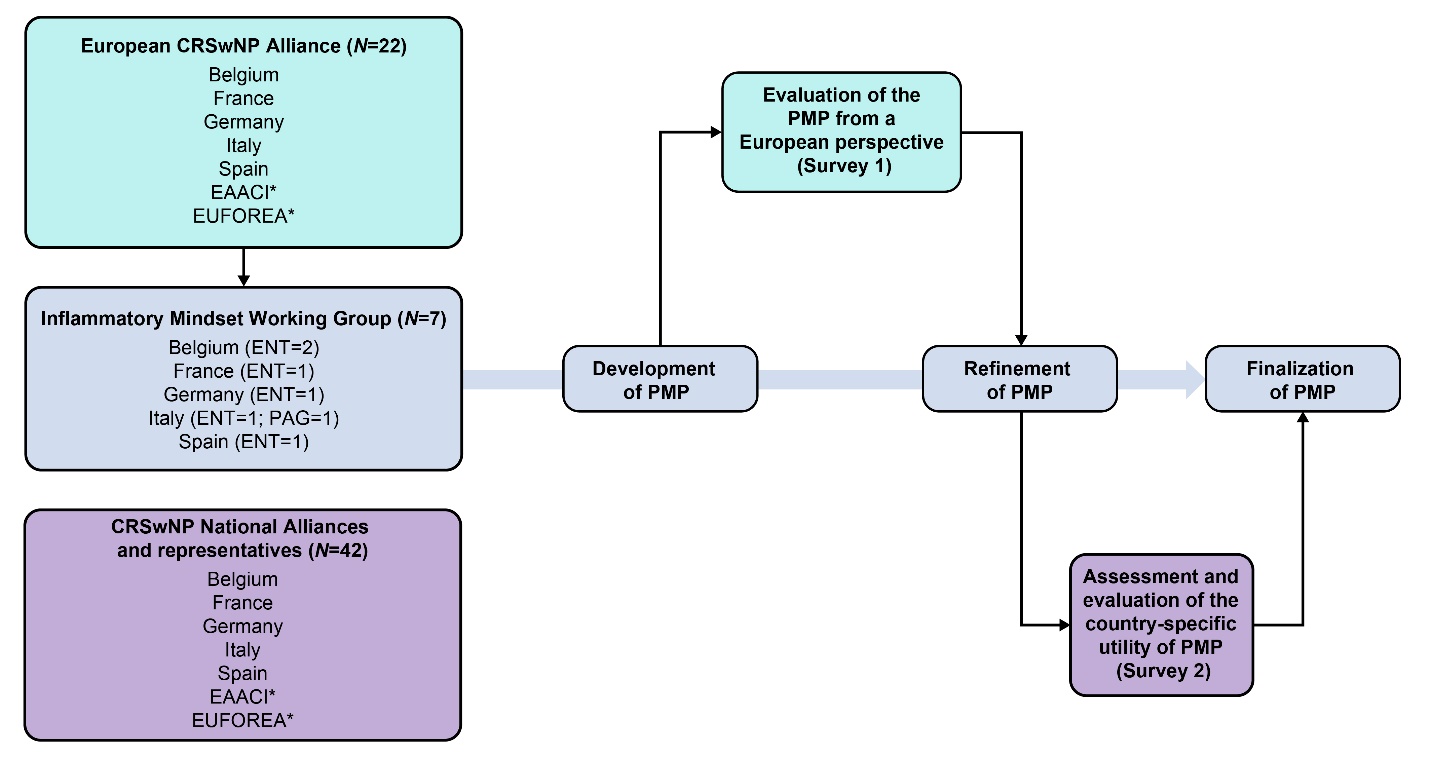
Otolaryngology specialists are referred to as ENT.
*Not part of the Alliance but partook in both Surveys.

CRSwNP, chronic rhinosinusitis with nasal polyps; EAACI, European Academy of Allergy & Clinical Immunology; EUFOREA, European Forum for Research and Education in Allergy and Airway Diseases; ENT, ear, nose, and throat; PAG, patient advocacy group; PMP, Patient Management Plan.

# Supplementary Fig. S3 Surveys 1 and 2: PMP feedback from HCP and PAG representatives on the type of support required to implement the proposed PMP into clinical practice


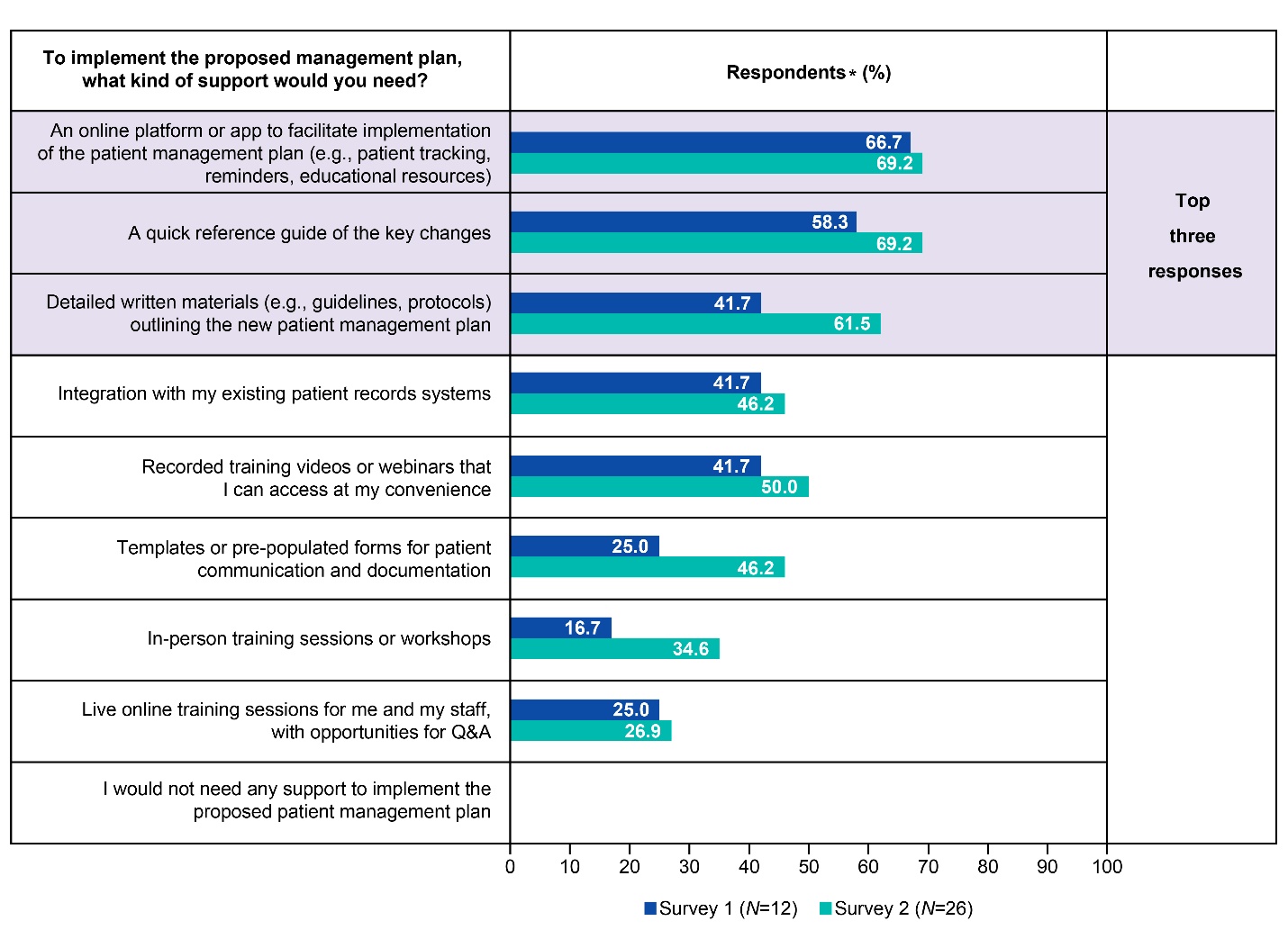


*Respondents were able to select more than one answer.
HCP, healthcare professional; PAG, patient advocacy group; PMP, Patient Management Plan; Q&A, questions and answers.
